# An analysis of specific batting demands in the women’s The Hundred competition

**DOI:** 10.17159/2078-516X/2023/v35i1a15056

**Published:** 2023-06-05

**Authors:** S Nicholls, J Keenan, AM Cresswell, L Pote

**Affiliations:** Department of Sport, Outdoor and Exercise Science, University of Derby, England

**Keywords:** The Hundred, batting, cricket, batters

## Abstract

**Background:**

No research has investigated the shortest format of the game of cricket, The Hundred competition. Furthermore, women’s cricket research is particularly limited, with most focusing on injuries and little literature investigating specific batting demands. These demands are important if training programmes are to mimic the game’s movement patterns.

**Objectives:**

The purpose of this study was to analyse specific batting demands and variables associated with the women’s The Hundred competition.

**Methods:**

Thirty-one matches from the Women’s 2021 The Hundred competition were analysed using Hudl Sportscode Elite. Variables analysed included: bowler type (seam or spin), free hits, no ball runs, reason for no ball (height/wide/front foot), run scored (0, 1, 2, 3, 4, 6), type of key event (fall of wicket, bowling referral, batting referral, umpire referral, bowling time out, rain delay, or injury) as well as time between deliveries and sets, overall and between the power play and non-power play. A total of 6073 deliveries were analysed.

**Results:**

A significant difference (p<0.05) was observed for time between deliveries for spin bowlers (26.90±22.16 s) compared to seam bowlers(31.70±20.37 s) as well as time between sets for the power play (58.00±13.28 s) and non-power play phases (63.70±42.00 s). Additionally, in the power play, most runs were made up of “1’s” and “4’s”. In the non-power play phase, “1’s” made up the biggest contribution of runs (as a percentage).

**Conclusion:**

The fact that singles make up a significant portion of a typical match means that strength and conditioning coaches should incorporate high-intensity sprint-type training into training programmes to mimic these demands.

The game of cricket was traditionally perceived to be a physically undemanding sport.^[1]^ However, over the decades, there has been a substantial amount of research showing that this is not the case, and cricketers need to be well-trained to compete.^[2,3]^ This is most likely due to the increased professionalism and evolution of the game from Test (multi-day) and one day matches to the T20 format. Cricket has therefore gone through several makeovers to keep up with the times^[4]^ and as a result, players are faced with shorter formats and packed schedules for their country, club, and franchise.

More recently, the newest, shortest, and fastest format of the game, The Hundred, has been introduced in the United Kingdom, where eight teams with players from all over the world compete in the five-week competition. A typical ‘Hundred’ match lasts approximately two and a half hours with each team facing 100 deliveries. Bowlers can deliver either five or ten consecutive balls (known as ‘sets’, instead of the traditional ‘overs’) with a maximum of 20 balls per bowler per innings (e.g. one set = five deliveries). The match outcome is ultimately decided by the team that scores the most runs. Similar to T20, the rules have been modified from traditional formats to provide an advantage to the batters.^[4]^ The first 25 balls constitute a power play where only two fielders are allowed outside the circle (27.43m area around the pitch), and ‘no balls’ award the batting side a ‘free hit’ plus two additional runs. Therefore, batting performance is an important aspect of the game and investigating the movement demands and physical requirements of batters is key for both coaching and strength and conditioning staff.^[5]^

Furthermore, one of the key drivers of developing the ‘The Hundred’ is the potential benefit to the women’s game, with increased visibility, investment, and exposure opportunities for players.^[6]^ The women’s game is growing, with 30% of cricketers overall and 60% of new cricketers in Australia being female;^[7]^ however, there is limited literature regarding the women’s cricket formats.^[8]^ A significant amount of research has performed time-motion analyses to quantify the movement patterns of male batters in Test (multi-day), one day and T20 matches.^[5,9]^ Findings indicate that the intensity of the game increases with the shorter formats and that batters perform at a similar intensity for one day matches and T20 matches respectively (Table 1).

Additionally, this similarity was also shown when comparing time between deliveries and overs for international one day and T20 matches. Average time between deliveries and overs for one day games was 32.70s and 79.80s respectively compared to 35.00s (between deliveries) and 75.00s (between overs) for T20 matches.^[2,10]^ Likewise, the running requirements of both formats of the game were shown to be similar in nature.^[2,10]^

While literature related to the movement demands for the multi-day, one day and T20 formats is substantially available, the same cannot be said for The Hundred competition. To the authors’ knowledge, these studies have also not considered all variables that could affect these movement patterns, such as power plays, bowler/batter referrals (to the third umpire), strategic time outs, rain delays and injury time, which are important if the actual demands of the game are to be shown. Furthermore, it has been indicated that these movement patterns are likely to differ between males and females because of factors such as anthropometry, force velocity relationships, boundary size, the size and mass of the ball, as well as the speed of the ball being bowled.^[11]^ Lastly, to date, no research has examined the movement demands of elite females in any of the match formats. The purpose of this study was therefore to analyse specific batting demands and variables associated with the women’s The Hundred competition. This is important so that trainers and coaches can design scientifically based programmes that mimic the demands of the female version of the game. In future, this could help with player performance and may reduce the risk of injury.

## Methods

### Sample

Thirty-four matches (32 regular seasons, one eliminator and one final) from the 2021 Women’s The Hundred competition were selected for analysis. The competition took place between 21^st^ July 2021 and 21^st^ August 2021. Three matches were excluded due to significant rain effects causing match abandonment (Manchester Originals v Northern Superchargers and Trent Rockets v Oval Invincibles) or video feed issues (Trent Rockets v Northern Superchargers) whereby match footage was unavailable. As a result, video footage from 31 of the collated matches was used for the final analysis. A total of 6073 deliveries were analysed.

### Variables and procedure

Match variables related to bowler type (seam or spin), whether it was a free hit, no ball runs (1, 2), no ball reason (height/wide/front foot), run scored (0, 1, 2, 3, 4, 6), and type of key event (fall of wicket, bowling referral, batting referral, umpire referral, bowling time out, rain delay, or injury) were selected. In addition, the time between deliveries (defined as the exact video frame immediately before ball release from the bowler’s hand), the time between sets (defined as the exact video frame between the umpire calling ‘set’ and the following ball delivery release frame), and the number of instances analysed were collected. Operational definitions for each variable were defined prior to the coding of match footage and were referenced during analysis to ensure consistency and accuracy of coding throughout.

To collate the data of interest, a bespoke panel was created within Hudl Sportscode Elite (Hudl, USA) to code each game and associated variables of interest. All data were manually coded by the same individual, visually reviewed, checked for entry and logic errors post-completion (e.g. if a wide was bowled then an extra delivery had been coded), and subsequently collated into a Microsoft Excel spreadsheet for further analysis.

### Reliability

Coded data were assessed for accuracy and reliability. In a similar manner to Kubayi and Larkin,^[12]^ data from approximately 10% of the matches analysed were randomly selected to be re-coded by the same observer following a minimum two-week separation to negate observer remembrance. Intra-rater reliability was assessed for each variable between the two coded timelines using a Pearson’s r coefficient (time between deliveries and time between overs) or kappa statistic (remaining coded variables). All variables demonstrated perfect (kappa = 1.00) or near perfect agreement (Pearson r > 0.99) and provided confidence that the collected data reliably represented observed match events.

### Statistical analysis

Descriptive data were presented as mean (SD) or relative contribution (%) where appropriate. Normality assumptions were checked using the Kolmogorov-Smirnov test. A Mann Whitney U test (IBM SPSS Statistics, Version 25) was used to identify differences in the overall time taken between deliveries in relation to delivery type (spin vs seam), whilst a Wilcoxon signed-rank test compared time between sets for power play and non-power play. Statistical significance was set at p < 0.05. The Friedman test was used to analyse the interaction between bowler type (seam/spin) and match period (powerplay/non-powerplay) in relation to time between deliveries. Post-hoc pairwise comparisons using the Mann Whitney U (Wilcoxon rank-sum) test with a Bonferroni correction were undertaken thereafter to minimise the risk of Type I errors due to multiplicity testing. The effect size calculation (Cohen’s *d*) was used to characterise the magnitude of difference for the time between deliveries and sets for the power play and the non-power play phases, for both seam and spin bowling.^[13]^ The criteria for interpreting effect sizes were: < 0.2 trivial, 0.2–0.5 small, > 0.5–0.8 medium, > 0.8 large. Corresponding 95% confidence intervals (95% CIs) were calculated.

## Results

A significant difference (p<0.001; ES=0.23) was observed for the time between deliveries for spin (26.90±22.16s; 95% CI: 26.11–27.66) compared to seam (31.70±20.37s; 95% CI: 30.94–32.42) bowlers overall. The Friedman test indicated a significant interaction between bowler type (seam/spin) and match period (power play/non-power play (X^2^(3)=97.35, p<0.001; ES=0.06). Significant differences were identified for the difference in time between spin and seam within both the power play (26.20±17.68; 95% CI: 24.44–27.90; and 31.30±19.06; 95% CI: 30.18–32.45; U=375016.500; p<0.001; ES=0.27) and the non-power play (27.00±22.52s; 95% CI: 26.17–27.89 and 31.90±21.12s; 95% CI: 30.93–32.87; U=3111791.000; p<0.001; ES=0.23) respectively (Table 2). No significance was shown within each discipline (spin and seam) for all phases of the game as well as time between sets.

Dot balls (‘0’s) and ‘1’s’ respectively constitute 39.16% and 39.22% of runs per delivery bowled (Table 3). In terms of run contribution ‘4’s’ make up the largest portion of runs scored in a women’s The Hundred match (45.64% of total runs scored).

Within the power play (first 25 deliveries), dot balls make up the majority of the deliveries bowled (54.0% contribution), followed by ‘1’s’ (25.1% contribution) and ‘4’s’ (16.5% contribution; Table 4). This changes outside of the power play (deliveries 26–100), where ‘1’s’ are more prevalent (44.3% contribution) followed by dot balls (33.8% contribution) and ‘4’s’ (12.7% contribution).

More seam deliveries are bowled in the power play phase of the game compared to spin (67.2% vs 32.8%; Table 5). The opposite is observed in the non-power play phase with seam deliveries making up 41.2% compared to 58.8% spin deliveries bowled. Relatively, more wickets fall (6.1% vs 4.6%) and there are more umpire referrals (1.2% vs 0.4%) in the non-power play phase compared to the powerplay. Contrastingly, more wides are bowled in the power play (5.4% vs 2.7% compared to the non-power play phase).

## Discussion

The most important finding of this investigation was that singles (1’s) made up the largest contribution (as a percentage) of running requirements for both the power play (25.1%) and non-power play (44.3%) phases of a women’s ‘Hundred’ match (Table 4). The same trend was observed overall, with 39.2% of running a result of singles (Table 3). The use of singles to accumulate winning totals is different to the observed trend in men’s short form games where winning totals are built on ‘4’s’; more boundaries are scored in the men’s format.^[14]^

Significant differences (p<0.01) were also observed for time between spin and seam time deliveries (26.90±22.16s vs 31.70±20.37s; ES=0.23), as well as time between spin and seam within the power play (27.00±22.52s vs 31.90±21.12s; ES=0.27), and non-power play (27.00±22.52s vs 31.90±21.12; ES=0.23) respectively. This is shorter than times observed for one day internationals (time between deliveries: 32.70s; time between overs: 79.80s) and T20 matches (time between deliveries: 35.00s; time between overs: 75.00s).^[2,10]^ This could be due to several factors. The rules are designed to encourage quicker play, bowlers do not change end after ever set and penalties are enforced for slow set rates in ‘The Hundred’ competition. If the bowling team does not complete their sets in an allocated time period, one fewer fielder will be allowed outside the 30-yard (27.43m) circle for the remaining sets, thus encouraging teams to ‘get through their sets’ quicker. In addition, the current study was on elite female cricketers while the studies investigated elite male players; more boundaries are hit during the men’s game perhaps resulting in a longer time to return the ball to the bowler. More boundaries are also hit in the men’s format of the game due to greater maximum bat speeds and ball launch speeds.^[11]^ Additionally, the boundary is smaller (closer) for the female game compared to the male setup, which could also account for the shorter times in this investigation.^[11]^

These findings create a unique insight into the Women’s ‘Hundred’ game. There is potentially a greater exposure to high-speed running for female batters than seen in their male counterparts. Furthermore, the reduced time between balls, and more significantly between sets, reduces the rest period and creates a higher density of action for the female players than observed in the men’s game. One of the key facets of successful batting performance is the accuracy and execution of skill, which is pressured in short forms of the game.^[15]^ If these factors are further pressured by an increased volume of workload and density of skill execution, batting performance could be impaired. Prolonged batting with repeated shuttle running has been reported to impact high-order cognitive function and therefore affect decision-making, response selection, response execution and other batting-related executive processes.^[16]^

These findings also support that the development of the players' sprint performance, and aerobic and anaerobic capacity will be vital to successful batting performance, this will facilitate both production of high intensity actions and recovery from these actions. Further to this, players should be exposed to sport-specific training that includes repeated shuttle running incorporating skill execution; however, this should be particular to the format being played. Batters are most prone to lower limb injuries (hamstring and quadriceps strains) because of high workloads and the high eccentric load due to the constant acceleration, deceleration and turning when sprinting between the wickets.^[17,18]^ Training plans for batters in the women’s ‘Hundred’ should also focus on high-velocity force application and the development of eccentric training regimes so that players can cope with the stress placed on them when sprinting between the wickets.^[3]^ This could improve overall performance, as well as reduce the risk of injury by delaying the onset of fatigue.^[3]^

For other key run-scoring metrics, more ‘4’s’ are scored during the power play compared to the non-power play phase; the opposite is seen for ‘6’s’ (Table 4). This is understandable as during the power play, more players need to be within the inner circle (27.43m) (30-yard circle) due to fielding restrictions and as a result more ‘4’s’ are hit. Furthermore, this is important as research on T20 matches has shown that winning teams hit more boundary ‘4’s’ in the power play (first six overs).^[14]^ More ‘6’s’ are hit in the non-power play as players often have to clear the boundary at the back end of an innings when the field is spread out (relaxed fielding restrictions); a trend that has been observed in T20 cricket.^[14]^ The same can be observed for running ‘2’s’; more are scored in the non-power play phase as the fielding restrictions have been lifted, which allows more opportunity for multiple runs.

Several interesting findings were also observed when examining the key events that take place in a match (Table 5). As a percentage of deliveries within the phase, more wickets are taken in the non-power play phase (6.1%) of the innings compared to the power play (4.6%). This is to be expected as either a team is being bowled out before the completion of the 100 balls, or batters are trying to ‘hit out’ and score as many runs towards the back end of the innings thus resulting in more wickets falling. The same trend is shown for umpire referrals; more are observed in the non-power play phase (1.2%) compared to the power play (0.4%). This is most likely because batters are trying to maximise non-boundary scoring opportunities (e.g., 1’s and 2’s) in the non-power play phase (as the field is spread out) which results in several run-out opportunities and hence umpire referrals. Furthermore, the fact that the wicket-keeper in the female format of the game is often standing up to the stumps (through observation), means that there are most likely more umpire referrals as a result of stumping chances. In terms of batting and bowling referrals, no difference was observed between the power play and non-power play phases. Seam deliveries make up 67.20% of deliveries in the power play compared to 32.9% spin deliveries; the opposite was observed in the non-power play phase (58.8% spin compared to 41.3% seam deliveries). This is similar to the literature examining professional T20 matches (Indian Premier League and English Domestic League) which showed that there is no advantage to using spin bowlers in the power play.^[19,20]^ It has been suggested that spin bowlers should be used in the middle overs to restrict the run rate when the field is spread out,^[18,19]^ which seems to be the case in the ‘Hundred’ as well. This could explain the increased amount of spin deliveries used in the non-power play phase. Lastly, more wide deliveries are bowled in the power play (5.4%) compared to the non-power play phase (2.7%). This could be due to the fact that more seam deliveries are bowled in the first 25 balls (power play) when the ball is new and swinging, compared to when the spinners bowl later on in the match.

### Practical applications and future research

This study is important for the design of scientifically based strength and conditioning programmes. It is important that training programmes both develop the specific bioenergetics required to support the movement demands of the game as well as sessions that simulate match situations as closely as possible to ensure that players are prepared for the unpredictable nature of the game of cricket and skill execution under fatigue. This is particularly important in this version of the women’s game as it appears to have demands of volume and density of workloads that are beyond those experienced by batters in the men’s game. This investigation could allow for the design of protocols that mimic certain phases of play for training purposes. Effectively designed programmes could also reduce the risk of injury and improve overall player performance and well-being. Taking the results of this investigation into account, most of the runs are scored through boundaries (‘4’s). Therefore, training should incorporate some form of range or power hitting. Upper body strength and power are other physical characteristics that need to be developed if the boundary is to be cleared more often. Furthermore, singles also contribute substantially to overall runs scored. As a result, training practices should focus on the rate of force development in the lower limbs to help with acceleration properties. This may similarly reduce the risk of hamstring strains due to the eccentric actions when accelerating, sprinting and decelerating while running between the wickets. Additionally, the information gained from this study can be used for the design of a ‘Hundred’ protocol that may be used for future research to take a more in depth look at the different responses of elite female cricketers. This could allow future research to examine, for example, the physiological and perceptual demands of the women’s ‘Hundred’. Lastly, a comparison between these findings and the male version of the competition is needed. Therefore, future research should consider replicating this investigation using male ‘Hundred’ cricketers to determine whether the movement demands and patterns differ between a male and female cohort.

## Conclusion

This is the first study to investigate the demands of the women’s “The Hundred” competition. The most significant finding was that singles (1’s) make up the majority of the running requirements, for both the power play and non-powerplay phases of a match. As a result, strength and conditioning coaches should focus on mimicking the demands of the game by incorporating high-intensity, shuttle running into their training programs. This may help with overall performance as well as reduce the risk of injury.

## Figures and Tables

**Table 1:** Movement variables of male batters for the different game formats (adapted from Petersen et al., 2010)^9^

	Sprints/hour (>5 m/s^−1^)	Mean sprint distance (m)	Maximum sprint distance (m)	Efforts/hour	Recovery ratio (1:*x*)
**Twenty20**	15 (9)	13 (4)	19 (6)	45 (16)	38 (13)
**One day**	13 (9)	11 (3)	21 (11)	39 (16)	50 (21)
**Multi-day**	8 (3)	13 (7)	21 (8)	28 (6)	61 (10)

Data expressed as mean (SD). “Efforts/hour”, number of movement efforts per hour; “Recovery ratio”, ratio between work performed and recovery time.

**Table 2:** Time between deliveries for spin and seam bowlers (overall, power play and non-power play) as well as time between sets (5 deliveries)

	Overall (s)	Power play (s)	Non-power play (s)
**Spin**	26.90 (22.16)*	26.20 (17.68) $	27.00 (22.52) #
**Seam**	31.70 (20.37)*	31.30 (19.06) $	31.90 (21.12) #
**Both**	29.20 (21.45)	29.60 (19.02)	29.00 (22.09)
**Between sets**	62.50 (37.71)	58.00 (13.28)	63.70 (42.00)

Data expressed as mean (SD).

*, #, $, represent significant difference between paired symbols. One set is 5 deliveries.

**Table 3:** Overall absolute runs and percentage run contribution for the 2021 Women’s The Hundred competition (n=31)

Run value	Number of deliveries	Delivery (%)	Total runs	Runsper set	Run (%)
**0**	2378	39.2	0	0.0	0.0
**1**	2382	39.2	2382	2.0	32.6
**2**	375	6.2	750	0.6	10.3
**3**	18	0.3	54	0.04	0.7
**4**	834	13.7	3336	2.8	45.6
**6**	86	1.4	516	0.4	7.1
**Extras** *	240	-	272	0.2	3.7

**Overall**	6073	100	7310	6.0	100

* Extras are additional runs (1 or 2) provided to the batting team because of an in-match event (wides = 1 run, no balls = 2 runs).

These extras do not form part of the total deliveries bowled by the fielding team. One set is 5 deliveries. Teams may be bowled out prior to completing their allotted 100 balls or 20 sets in a match, or targets are chased down with balls/sets in hand.

**Table 4:** Absolute and percentage (%) of deliveries and run contribution in the power play compared to the non-power play phase of the 2021 Women’s The Hundred competition (n=31)

	Power play					Non-power play				

Run value	Number of deliveries	Delivery (%)	Total Runs	Runs per set	Run(%)	Number of deliveries	Delivery (%)	Total Runs	Runsper set	Run (%)
**0**	874	54.0	0	0	0.0	1506	33.8	0	0	0
**1**	406	25.1	406	1.3	22.8	1974	44.3	1974	2.2	35.7
**2**	56	3.5	112	0.4	6.3	319	7.2	638	0.7	11.5
**3**	3	0.2	9	0.03	0.5	15	0.3	45	0.05	0.8
**4**	267	16.5	1068	3.3	60.0	567	12.7	2268	2.6	41.0
**6**	13	0.8	78	0.2	4.4	73	1.6	438	0.5	7.9
**Extras** *	98	-	108	0.3	6.1	142	-	164	0.2	3.0

**Overall**	1619	100	1781	5.5	100	4454	100	5527	6.3	100

* Extras are additional runs (1 or 2) provided to the batting team because of an in-match event (wides = 1 run, no balls = 2 runs).

These extras do not form part of the total deliveries bowled by the fielding team. “Power-play” refers to the first 25 deliveries and “Non-power play” is the remaining 75 deliveries in an innings. One set is 5 deliveries. Teams may be bowled out prior to completing their allotted 100 balls or 20 sets in a match, or targets are chased down with balls/sets in hand.

**Table 5:** Total, percentage (of overall balls bowled) and per game key events in the power play compared to the non-power play phase of the 2021 Women’s The Hundred competition (n=31)

Key Event	Power play			Non-power play		

	Total number	Percentage of total deliveries (%)	Number per game	Total number	Percentage of total deliveries (%)	Number per game
**Fall of wicket**	75	4.6	2.4	270	6.1	8.7
**Umpire referral**	6	0.4	0.2	52	1.2	1.9
**Bowling referral**	8	0.5	0.3	26	0.6	0.8
**Batting referral**	3	0.2	0.1	11	0.2	0.4
**Injury**	1	0.1	0.03	2	0.0	0.1
**Bowling time out**	0	0.0	0.0	45	1.0	1.5
**Free hits**	5	0.3	0.2	20	0.4	0.7
**No balls**	10	0.6	0.3	22	0.5	0.7
**Wides**	88	5.4	2.8	119	2.7	3.8

**Seam deliveries**	1088	67.2	35.1	1833	41.2	59.1
**Spin deliveries**	531	32.8	17.1	2621	58.8	84.6

“Power-play” refers to the first 25 deliveries and “Non-power play” is the remaining 75 deliveries in an innings. Seam and Spin deliveries refer to all key events, but exclude wides, no balls, and free hits. One set is 5 deliveries. Teams may be bowled out prior to completing their allotted 100 balls or 20 sets in a match, or targets are chased down with balls/sets in hand.
